# Photosynthetic Systems Suggest an Evolutionary Pathway to Diderms

**DOI:** 10.1007/s10441-020-09402-y

**Published:** 2020-12-07

**Authors:** Scott O. Rogers

**Affiliations:** Department of Biological Sciences, Bowling Green State University, Bowling Green, OH 43403 USA

**Keywords:** Monoderms, Diderms, Gram-positive, Gram-negative, Photosynthesis, Endosymbiotic

## Abstract

Bacteria are divided primarily into monoderms (with one cell membrane, and usually Gram-positive, due to a thick peptidoglycan layer) and diderms (with two cell membranes, and mostly Gram-negative, due to a thin peptidoglycan layer sandwiched between the two membranes). Photosynthetic species are spread among the taxonomic groups, some having type I reaction centers (RCI in monoderm phylum Firmicutes; and diderm phyla Acidobacteria and Chlorobi), others with type II reaction centers (RCII in monoderm phylum Chloroflexi; and diderm taxa Gemmatimonadetes, and alpha-, beta-, and gamma-Proteobacteria), and some containing both (RCI and RCII, only in diderm phylum Cyanobacteria). In most bacterial phylograms, photosystem types and diderm taxa are polyphyletic. A more parsimonious arrangement, which is supported by photosystem evolution, as well as additional sets of molecular characters, suggests that endosymbiotic events resulted in the formation of the diderms. In the model presented, monoderms readily form a monophyletic group, while diderms are produced by at least two endosymbiotic events, followed by additional evolutionary changes.

## Introduction

The search for a universal tree of life, especially one that resolves the earlier events in biological evolution, has been confounded by a number of factors, including the antiquities of branch points, the absence of sequence data from extinct organisms, rapid mutation rates (especially in the early stages), and horizontal (or lateral) gene transfers (HGTs). These factors lead to discordant placements and timings for branch points on the trees, resulting in confusion regarding the evolutionary events that produced the myriad taxa. HGTs are common and widespread within and among all domains of life (Archibald and Keeling 2002; Bowler et al. 2009; Dagan et al. 2008; Gao and Gupta 2012; Jain et al. 2002; Keeling 2010; Ku et al. 2015; Lake and Rivera 1994; Lang and Gray 1999; Lang et al. 2012; Martin and Muller 1998; Poole et al. 1999; Raymond et al. 2002; Sousy et al. 2015; Syvanen and Kado 1998). They can be caused by transfer of DNA from one organism to another, importation of DNA (within a food organism or freely floating), transfer by viruses, or by endosymbiotic events. In particular, endosymbiosis can transfer large numbers of genes in a single event, and have led to acquisition of complex functions (e.g., oxidative phosphorylation in eukaryotes and photosynthesis in plants). Endosymbioses led to the origin of eukaryotes, which joined at least one bacterium with an archaeal cell (Eme et al. 2017; Sagan 1967), and have resulted in the evolution of many types of organelles, including mitochondria, plastids, and chromatophores, as well as a variety of other complex organelles in protists. Because many of these events have involved bacteria, it is likely that bacteria (and possibly archaea) have also participated in other such events that have been obscured by mutations and genetic rearrangements over extended periods of time. Ancient endosymbiotic events have been proposed to account for some of the major HGTs in bacteria (involving dozens to hundreds of genes), but these have yet to be widely accepted (Gupta 2010; Lake 2009; Lang et al. 2012; Overmann and Schubert 2002).

Arguments can be made in favor of endosymbiotic events that led to diderms, using the molecular characters of photosystems (reaction centers RCI and RCII: and photosystems PSI and PSII), and other systems (e.g., nitrogen fixation and membrane characteristics). The reaction centers and photosystems are produced from large gene clusters, consisting of dozens of genes that are controlled by large numbers of transcription factors. Many additional gene products produce chlorophylls and other attendant molecules. Therefore, it is unlikely that such coordinated large and complex gene clusters have been successfully horizontally transferred *in toto* by simple HGTs. Furthermore, if small pieces of the clusters were transferred separately, which is theoretically possible, integration and coordinated regulation of the genes, and accurate function of the gene products, are improbable. It is more likely that they were inherited *en masse*, for example via endosymbiotic events, where the gene clusters were introduced as complete (or nearly complete) functional clusters.

**Fig. 1:**
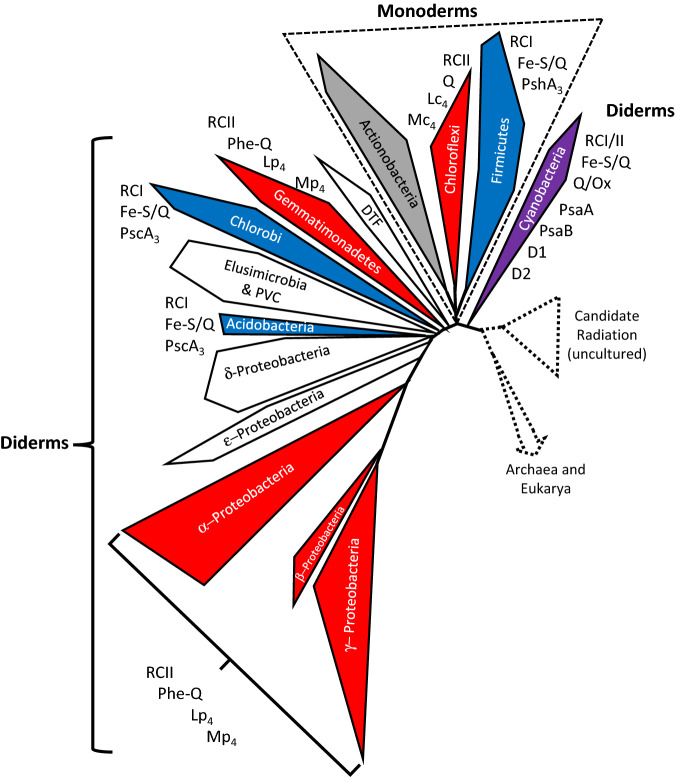
Phylogram of Bacteria (based on a condensed version of the phylogram in Hug et al. (2016), excluding the Candidate Radiation, Archaea, and Eukarya clades, in dashed lines). The original phylogram includes sequences from more than 3000 genomes from over 120 phyla (92 bacterial phyla). Monoderms, including the Actinobacteria (gray), Chloroflexi (red), and Firmicutes (blue) clades form a monophyletic group (outlined with dashed triangle), but diderms are polyphyletic, with the Cyanobacteria (violet) being separated from all other diderms. Photosystems also are polyphyletic, with some phyla with only RCI (Acidobacteria and Chlorobi -blue) and others with only RCII (Gemmatimonadetes and Proteobacteria-red). Members of Cyanobacteria (violet) have both RCI and RCII. The types of photosystems: Fe-S/Q, Q, and Phe-Q, also are shown

Monoderm bacteria (most of which are Gram-positive) are surrounded by a single cell membrane and comprise a basal group of bacteria that most often are monophyletic in phylogenetic and phyletic analyses (Fig. 1; see e.g., Hug et al. 2016). Diderms (most of which are Gram-negative) are surrounded by two distinct membranes, including some taxa with outer membranes that differ from other diderm outer membranes, indicating some heterogeneity within this group. In contrast to monoderms, diderms are consistently polyphyletic, being split by one or more monoderm phyla, and often existing on widely separated branches of phylograms (Ciccarelli et al. 2006; Forterre 2015; Hug et al. 2016; Marin et al. 2016; Satoh et al. 2013). While most of the diderms usually branch off far from most monoderms (with the exception of a few of the most basal phyla), one major phylum (Cyanobacteria) often branches off closest to the monoderms and is frequently separated from all other diderms in phylograms (Fig. 1). Another unique feature of Cyanobacteria is that most members have both photosystem I (with RCI) and photosystem II (with RCII), while all other photosynthetic diderms have only RCI or RCII. This study was initiated to investigate why Cyanobacteria is unique among diderms, and more generally to determine whether endosymbiotic events might explain the origins of this diderm phylum, as well as others.

## Materials and Methods

**Fig. 2:**
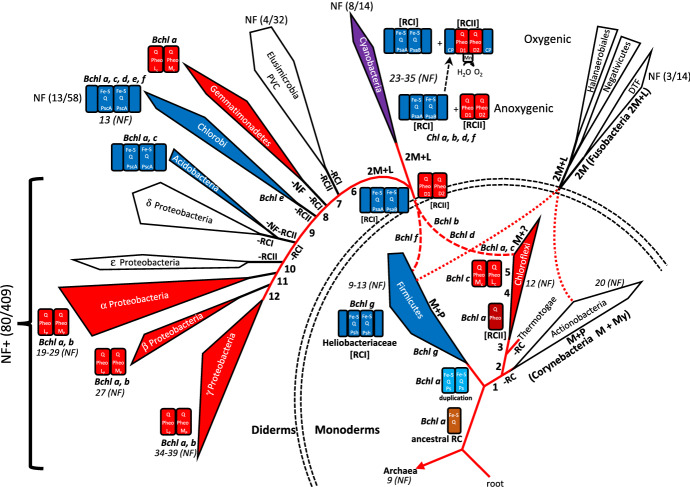
Clades from Fig. 1 reorganized with two endosymbiotic events between monoderms leading to diderms. The reorganization is based primarily on parsimonious changes for photosystems and other characteristics (Tables 1 and 2). This model increases parsimony and reduces polyphyly of membrane number, membrane types, amino acid changes, and photosystems. The monoderm phyla remain monophyletic (lower right), and are basal in the phylogram. Phylogenetic analyses based on 16S rRNA sequences support this organization, indicating that members of Chloroflexi (with RCII) and Heliobacteriaceae (Firmicutes, with RCI) were among the first photosynthetic phyla to appear in Domain Bacteria. The ancestral photosystem (brown rectangle) predated the separation of Chloroflexi, Firmicutes, and Actinobacteria (Blankenship 1992; Jain et al. 1999; Satoh et al. 2013; Woese 1987). Diderms fall into four general groups, those that have only RCI (blue), only RC II (red), both RCI and RCII (violet), and those that lack photosynthesis of any kind (white). Numbers (1–12) superimposed on the phylogram are the mutations reported by others (Antunes et al. 2016; Gupta 2003, 2011; Imaeda et al. 1968) among the Bacteria (details in Table 2). Membrane types are indicated as follows: M + P = single membrane with peptidoglycan layer; M + Myc = single membrane with mycolic acid layer; M+? = single membrane with uncharacterized outer layer; 2M = double membrane; 2M + L = double membrane with lipopolysaccharides in the outer membrane. Bacteriochlorophylls (*Bcl*) and chlorophylls (*Chl*) are noted where they first appear in evolution. Nitrogen fixation is noted on the phylogram. Numbers in parentheses indicate the number of taxa that are capable of nitrogen fixation over the total number of taxa examined in those phyla. Italicized numbers followed by (*NF*) indicate the number of genes for nitrogen fixation in members of those taxa. The number of nitrogen fixation genes rapidly increases from monoderms to diderms, suggesting the possible addition of genes from the two members participating in the endosymbiotic event. Losses of function are indicated by -NF, -RCI, or -RCII

Several phylogenetic trees were used as the starting point for grouping of bacterial taxa (Cardona 2015, 2016a, b, 2018; Ciccarelli et al. 2006; Forterre 2015; Goldfarb et al. 2011; Hug et al. 2016; Lebedinsky et al. 2007; Macalady et al. 2013; Marin et al. 2016; Pace et al. 2012; Sousa et al. 2012; Tebo et al. 2015). The characteristics of photosystems among the bacteria were then mapped onto a condensed version of one of the most comprehensive trees (Fig. 1; Hug et al. 2016). Photosystems were polyphyletic. Some taxonomic groups had only photosynthetic systems related to RCI, including some members of Frimicutes (monoderm), as well as Acidobacteria and Chlorobi (both diderms). Others had only photosynthetic systems related to RCII, including Chloroflexi (monoderm), as well as diderms Gemmatimonadetes and several proteobacterial taxa (alpha-, beta-, and gamma-Proteobacteria). Only one diderm phylum, Cyanobacteria, had both RCI and RCII, and was closest to Firmicutes in the tree, and in most phylogenetic trees. Several other published phylogenetic trees were then used to determine the closest sister phyla to the phylum that contained photosynthetic members (Table 1). The original phylogram (Fig. 1) was then revised, based on sister taxa, while considering potential endosymbiotic events leading to diderm membrane systems (Fig. 2). Combinations of two of each of the monoderm phyla (Firmicutes + Chloroflexi; Chloroflexi + Actinobacteria; and Firmicutes + Actinobacteria) were examined to determine the most parsimonious combinations for endosymbiotic events. Subsequently, amino acid insertions and deletions in several proteins (Table 2) reported by Gupta (1998, 2003, 2010) were mapped onto the resulting phylogram to determine whether the tree was consistent with these changes (Fig. 2). Gene clusters for nitrogen fixation and membrane characteristics also were mapped onto the tree (Yan et al. 2008; Wang et al. 2013). Therefore, the final phylogram and proposed endosymbiotic events considers the origin of the major taxonomic groups, shared mutations, numbers of genes for nitrogen fixation, and membrane and photosystem characteristics.

**Table 1:** Enumerations of associations among phyla to sister photosynthetic phyla. Primary sister taxa are based on the number of phylograms where the two phyla are adjacent. Other related taxa are close, but not adjacent

Phylum^a^	Primary sister taxa^b^	Other related taxa^b^
[Actinobacteria]	Chloroflexi (3), **[DTF]**^**c**^ **(3)**, Firmicutes (4)	**Acidobacteria (1), Chlorobi (1)**, **Cyanobacteria (2), [PVC]**^**d**^ **(1)**
Chloroflexi	**Cyanobacteria (5), [DTF] (6)**, Firmicutes (4)	**Acidobacteria (2)**, [Actinobacteria] (2), **Proteobacteria (2)**
Firmicutes	[Actinobacteria] (4), Chloroflexi (4), **Cyanobacteria (6)**, **[DTF] (3)**	**Acidobacteria (2), Chlorobi (2), Proteobacteria (1)**
**Acidobacteria**	**Proteobacteria (3)**	[Actinobacteria] (1), **Chlorobi (1)**, [**DTF] (2)**
**Gemmatimonadetes**	**Chlorobi (4)**	**[**DTF] **(2), Proteobacteria (2)**
**Chlorobi**	**Acidobacteria (3)**, **[Bacteroidetes] (6)**, **Gemmatimonadetes/Fibrobacteres (3)**, **Proteobacteria (6)**	Chloroflexi (1), Firmicutes (1), **[PVC] (1)**
**Cyanobacteria**	[Actinobacteria] (3), Chloroflexi (9), Firmicutes (6)	**Acidobacteria (1)**, **Chlorobi (1)**, [**DTF] (1), Proteobacteria (2)**
**Proteobacteria (α, β, γ)**	**Acidobacteria (7)**, **[Bacteroidetes] (3), Chlorobi (3)**, Chloroflexi (3), **Cyanobacteria (4)**	[Actinobacteria] (1), Firmicutes (1), [**DTF] (1), [PVC] (1)**

^a^Monoderm phyla are in normal font, diderms are in bold font, non-photosynthetic phyla are in brackets

^b^Numbers in parentheses indicate the number of associations (adjacent sister taxon, or taxa that are close but not adjacent) in the referenced phylograms (Cardona 2015, 2016a, b, 2018; Ciccarelli et al. 2006; Forterre 2015; Goldfarb et al. 2011; Hug et al. 2016; Lebedinsky et al. 2007; Macalady et al. 2013; Marin et al. 2016; Pace et al. 2012; Sousa et al. 2012; Tebo et al. 2015)

^c^DTF = *Deinococcus*, *Thermus*, Fusobacteria and related taxa

^d^ PVC = Planctomycete, Verrumicrobia, and Chlamydiae

**Table 2:** List of successive mutations among bacterial (based on Gupta 2003)

Group^a^	Mutation(s)	Present in Taxon (Taxa)^b^
1	13 amino acid (aa) deletion in ribosomal S12 protein	Acid, Act, Alpha, Beta, Chlb, Chlf, Cyan, Delta, DTF, Elus, Epsi, Gamma, Gem, Hal, Neg, PVC, Ther
2	3–4 aa deletion in SecF protein, 15–17 aa deletion in chorismate synthase	Acid, Alpha, Beta, Chlb, Chlf, Cyan, Delta, Elus, Epsi, Gamma, Gem, PVC, Ther
3	21–23 aa insertion in Hsp70 protein, 5 aa deletion in Hsp90 protein	Acid, Alpha, Beta, Chlb, Chlf, Cyan, Delta, Elus, Epsi, Gamma, Gem, PVC
4	2 aa deletion in chorismate synthase	Acid, Alpha, Beta, Chlb, Chlf, Cyan, Delta, Elus, Epsi, Gamma, Gem, PVC
5	> 150 aa insertion in RNA polymerase β’, 1 aa insertion in Hsp60 protein	Acid, Alpha, Beta, Chlb, Chlf, Cyan, Delta, Elus, Epsi, Gamma, Gem, PVC
6	1 aa insertion in FtsZ protein, 2 aa insertion in Rho protein	Acid, Alpha, Beta, Chlb, Delta, Elus, Epsi, Gamma, Gem, PVC
7	100–120 aa insertion in RNA polymerase β, 4 aa insertion in alanyl-tRNA synthase	Acid, Alpha, Beta, Chlb, Delta, Epsi, Gamma, Gem
8	2 aa insertion in inorganic pyrophosphatase	Acid, Alpha, Beta, Chlb, Delta, Epsi, Gamma
9	2 aa insertion in Hsp70, 10 aa insert in CTP synthase	Acid, Alpha, Beta, Delta, Epsi, Gamma
10	1 aa deletion in Lon protease, 7 aa insertion in Sec A protein, 26–34 aa insertion in gyrase A, 3 aa insert in Rho protein	Alpha, Beta, Gamma
11	4 aa insert in Hsp70, 37 aa insert in valyl-tRNA synthetase, 1 aa insert in PRPP phosphoribosylpyrophosphate synthetase, 1 aa insert in biotin carboxylase, 11 aa insert in ATP synthase α subunit	Beta, Gamma
12	2 aa deletion in PAC-transformylase	Gamma

^a^Mutation group numbers correspond to those numerals on the phylogram (Fig. 2) and in the pathway (Fig. 3)

^b^Abbreviations: Includes: Acid = Acidobacteria; Act = Actinobacteria; Alpha = Alphaproteobacteria; Beta = Betaproteobacteria; Chlb = Chlorobi; Chlf = Chloroflexi; Cyan = Cyanobacteria; Delta = Deltaproteobacteria; DTF = Deinococcales, Thermus, Fusobacteria; Elus = Elusimicrobia; Epsi = Epsilonproteobacteria; Gamma = Gammaproteobacteria; Gem = Gemmatimonadetes; Hal = Halanaerobiales; Neg = Negativicutes; PVC = Planctomycetes, Verrumicrobia, Chlamidiae; Ther = Thermotogales. [Note: None of the mutations occurs in Firmicutes]

## Results and Discussion

### Overall Placement of Taxa

The phylogram in Fig. 1 is a condensed version from Hug et al. (2016), based on sequences from more than 3000 organisms, including those from 92 bacterial phyla. It indicates that membrane and photosynthetic systems are polyphyletic. Both are reliant on hundreds of gene products. This combination of polyphyly and the involvement of hundreds of coordinately expressed genes is inconsistent with solely vertical inheritance of those systems. Additionally, Cyanobacteria contains RCI and RCII, which evolved separately before joining in the ancestors of this phylum. In Fig. 2, each of the clades of the phylogram (in Fig. 1) have been maintained, but the branches joining each of the clades have been altered in order to join the clades according to the proposed endosymbiotic events, photosystem types, and homologous protein characteristics. At least two independent endosymbiotic events were needed to construct a tree that produced all monophyletic phyla and had a minimum of other changes. While the main group is formed by the majority of the diderm phyla, all having amino acid substitutions 1–5 (Table 2; Fig. 2), in order to join the Negativicutes, Halanaerobiales, and the DTF group (Deinococcus, Thermus, Fusobacteria), a second endosymbiotic event was necessary, because they have only substitution 1.

### Diderms and Monoderms

Diderms are of several types. The main phyla (e.g. all of the Proteobacteria, Bacteroidetes, Chlorobi, and Gemmatimonades) have an inner membrane, a thin peptidoglycan layer, and an outer membrane containing lipopolysaccharides. However, some taxa have thicker layers of peptidoglycan, also with outer membranes containing lipopolysaccharides (e.g., Negativicutes, Elusimicrobia, Fusobacteria, and Synergistetes) or are devoid of lipopolysaccharides (*Deinococcus*-*Thermus*), indicating some heterogeneity among the diderms. While Cyanobacteria often are included with the main diderm group, the phylogenetic position, photosystems, and other molecular characteristics often indicate that it is separated from other diderms, and has affinities to monoderms (Fig. 2; Table 1).

Monoderms have a single membrane and a thick peptidoglycan layer. Some have additional components to their outer layers (e.g., a mycolic acid layer in *Corynebacteria*), but lack a second membrane. Contrasting with diderms, monoderm phyla are almost always monophyletic in phylogenetic analyses. Members of one phylum, Thermotogae, have a distinctive outer membrane that consists of mainly protein, and have been difficult to position in many phylogenetic analyses. They are usually considered to be monoderms, but are sometimes considered atypical diderms. Their most parsimonious position in the phylogram (Fig. 2) also is among the monoderm clades, based primarily on the conserved amino acid changes among all bacterial groups included.

### Possible Scenarios of Diderm Evolution

There are four possible evolutionary pathways that can lead to the polyphyletic patterns outlined above: (1) The second membrane in diderms arose several times *de novo* without any HGTs; (2) Diderms originated from a monoderm taxon after one or more large HGTs of genes responsible for membrane and cell wall structure; (3) Monoderms arose from diderms by the reduction of one membrane; or (4) Diderms originated from one or more endosymbiotic events between two monoderms. The first possibility would require several rounds of duplications of dozens to hundreds of genes, followed by divergence, and accurate export of materials to construct the outer membrane. While possible, and many genes have evolved via gene duplication and divergence, this would not explain the presence of RCI (PSI) and RCII (PSII) in Cyanobacteria, because studies have shown that these two systems evolved separately in other organisms prior to appearing together in Cyanobacteria (Beanland 1990; Cardona 2015, 2016a, b, 2018; Mulkidjanian and Junge 1997; Mulkidjanian et al. 2005). Also, the fact that the generation of the second membrane would have to have occurred multiple times in separate taxa renders this scenario unlikely. The second possible origin for the two membrane systems involves the importation of hundreds of genes that carried the instructions for construction of the outer membrane lipids, proteins, saccharides, and polysaccharides. While this is possible, and there are HGTs where multiple genes have been transferred, an HGT consisting of dozens to hundreds of genes, all of which are able to be properly expressed in a new organism, has a low probability (Bowler 2009; Jain et al. 1999). A similar argument can be made for acquisition of photosystem genes, especially the presence of both photosystems in Cyanobacteria.

The third possibility presents a non-parsimonious outcome, because it implies that diderms evolved first (which is not supported by most phylogenetic studies), and that monoderms were derived from these by loss of genes and membranes. It fails to address how and when a two-membrane system first appeared that predated a single-membrane system (which requires fewer genes and gene products). A more parsimonious solution is to begin with a single membrane, and then build a second membrane system from a second set of genes (or duplication of an existing set) and gene products.

It is proposed here that the fourth possibility is the most plausible, and the most parsimonious, with respect to the number of events and genes required. Additionally, 16S rRNA phylogenetic analyses indicate that phylum Chloroflexi was among the first photosynthetic organisms on Earth, followed by the Heliobacteriaceae (within phylum Firmicutes), and only later did the photosynthetic phyla Chlorobi, Proteobacteria, Cyanobacteria, and others appear (Blankenship 1992; Satoh et al. 2013; Woese 1987). While all known members of Actinobacteria (a monoderm phylum) are incapable of photosynthesis, some genes related to photosynthetic processes have been reported in the genomes of some members, indicating an early loss of photosynthesis in this phylum, and supporting an ancient origin of photosynthesis (Gupta and Khadka 2016). This is consistent with the fourth possibility and is also consistent with extensive phylogenetic analyses based on genomic sequence data from more than 3000 organisms, including those from 92 bacterial phyla (Hug et al. 2016).

### Phylogenies of Cyanobacteria and Photosynthetic Systems

**Fig. 3:**
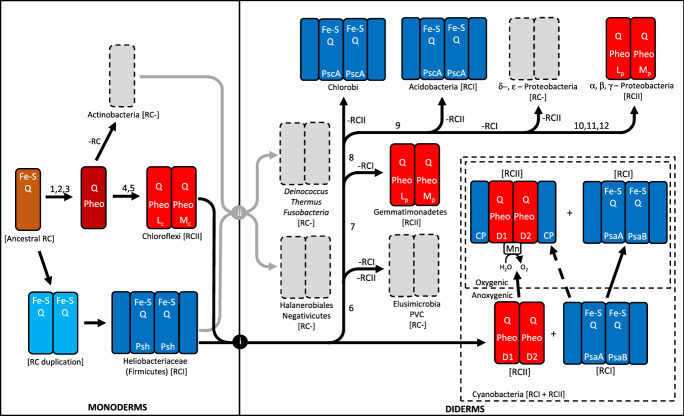
Model of the evolution of monoderms (left) and diderms (right) with emphasis on parsimony of photosystems. All of the reaction centers and photosystems emerged from an ancestral reaction center (brown rectangle, left) more than 3.0 billion years ago (Beanland 1990; Blankenship 1992; Cardona 2015, 2016a, b, 2018; Mulkidjanian and Junge 1997; Mulkidjanian et al. 2005). The first photosystems were based on iron-sulfur (Fe-S) and quinone (Q) reaction centers. Next, there was a duplication and diversification of the reaction center. During diversification of the monoderms, three major clades emerged: Heliobacteriaceae (within the Firmicutes), which retained an Fe-S Q photosystem (RCI, light blue and blue rectangles); Chloroflexi, which includes many photosynthetic species, all of which use a pheophytin quinone (Pheo Q) reaction center (RCII), where the two subunits have diverged into L and M types (red rectangles); and Actinobacteria, some members of which have retained some photosynthesis genes (Gupta and Khadka 2016), but the phylum is devoid of photosynthetic species (grey). Most Firmicutes (except the Heliobacteriaceae) have lost their photosynthetic capabilities. One of the endosymbiotic events (i - black circle) led to the Cyanobacteria, as well as most of the other diderms. The other endosymbiotic event (ii - grey circle) led to the *Deinococcus*-*Thermus-*Fusobacteria clade and the Halanerobiales and Negativicutes clades. In many taxa, photosynthetic capabilities were lost (gray rectangles). In a few phyla, only one of the two reaction centers was retained (RCI in Chlorobi and Acidobacteria; and RCII in Gemmatimonadetes and alpha-, beta-, and gamma-Proteobacteria). Cyanobacteria retained both photosystems, combining parts of the photosystems, and gained the ability to generate oxygen, through the addition of a manganese (Mn) moiety. Numerals on some of the branches indicate the amino acid changes detailed in Table 2, and shown in Fig. 2. For the final branch, a-proteobacteria have only mutation 10, b-proteobacteria have mutation 10 and 11, and gamma-proteobacteria have 10, 11, and 12

In most phylogenetic analyses, the phylum Cyanobacteria is isolated from all or most other diderm phyla, making the diderms polyphyletic, both in terms of membrane structure and photosynthesis types (Blankenship 1992; Hug et al. 2016; Woese 1987). One hypothesis proposed previously for the origination of diderms is that of an endosymbiotic event more than 3 billion years ago between a member of Actinobacteria (non-photosynthetic) and a member of Firmicutes (a Clostridia, also non-photosynthetic), both of which are monoderms (Lake 2009; and discussed in Gupta and Khadka 2016). This is phylogenetically consistent for some diderms, but Cyanobacteria clusters closer to Firmicutes and Chloroflexi than to Actinobacteria and the major groups of diderms, indicating that a single event (endosymbiotic or otherwise) involving a member of the Actinobacteria and another from the Firmicutes is insufficient to explain the evolution of all diderms. While the previously proposed endosymbiotic model for the origin of diderms is plausible (Lake 2009), the choice of genomes failed to account for photosystems and their polyphyly. Furthermore, an argument described by Gupta (2011) provides a convincing case against this specific hypothesis. The only members of the Firmicutes that are known to be photosynthetic are within the family Heliobacteriaceae, whose members carry genes only for RCI, while most diderms, including Proteobacteria have only RCII. A more parsimonious solution is that a member of Chloroflexi (monoderms that have only RCII) joined with another monoderm, likely from the Heliobacteriaceae (phylum Firmicutes) that has only RCI, in an endosymbiotic event to initiate the majority of the diderms. This resolves the polyphyly between most monoderms and diderms, and also resolves the phylogenetic placement of Cyanobacteria with its two photosystems (Figs. 2 and 3). Most of the other diderm phyla would then have lost either one (loss of RCI in Gemmatimonadetes, and alpha-, beta-, and gamma-Proteobacteria; and loss of RCII in Chlorobi and Acidobacteria) or both of the photosystems (in Elusimicrobia, and delta- and epsilon-Proteobacteria). Because each of these relies on dozens of genes for their functions, the probability of their loss is high due to mutation in one or more genes. Beyond inactivation by mutation, incompatibilities in gene and gene product interactions could cause failure of one or both of the photosystems (Beanland 1990; Blankenship 1992; Cardona 2015, 2016a, b, 2018; Gupta 2010; Mulkidjanian and Junge 1997; Mulkidjanian et al. 2005; Overmann and Schubert 2002; Zeng et al. 2014).

### Amino Acid Substitutions and Endosymbiotic Origin of Diderms

Studies of various genes and mutations have led to the analyses of a set of deletions and insertions of amino acids among proteins in a collection of bacterial taxa (Gupta 2003, 2010; Table 2). These have been mapped onto the phylogram (Fig. 2) and the proposed pathway (Fig. 3). While the earliest events (including deletions in ribosomal protein S12, SecF protein, chorismate synthase, and Hsp90; and insertions in Hsp70, RNA polymerase beta’, and Hsp60) are consistent with a single endosymbiotic event to lead to most diderms, it is inconsistent with the placements of the clades consisting of *Deinococcus*, *Thermus*, Fusobacteria, Negativicutes, and Halanerobiales. At least one additional endosymbiotic event would have to have occurred (Figs. 2 and 3). This second event is most parsimonious with an endosymbiotic event between a member of the Firmicutes and another from Actinobacteria (similar to that proposed by Lake 2009) to produce the *Dinococcus*-*Thermus-*Fusobacteria clade, and the Halanerobiales and Negativicutes clade (all non-photosynthetic). This is also consistent with cell wall and membrane evolution outlined by Gupta (2003, 2010), chlorophyll type, and nitrogen fixation, both of which have also been mapped onto the phylogram (Fig. 2). Placements of each of these taxa have been problematic in the past. One reason for this might be that they were the result of separate evolutionary pathways that led to similar morphologies, and vertical inheritance was assumed to be the primary mode of evolution.

### Origin of The Two Photosystems in Cyanobacteria

One of the major questions that has yet to be adequately answered is how did Cyanobacteria acquire both RCI and RCII, each reliant on the coordinated functions of dozens to more than 100 genes and gene products. Although the two photosystems probably diverged from a single ancestral form (Figs. 2 and 3) more than 3 billion years ago, the two contemporary photosystems differ significantly in their wavelength optima, and their basic functions and components (Beanland 1990; Blankenship 1992; Cardona 2015, 2016a, b, 2018; Mulkidjanian and Junge 1997; Mulkidjanian et al. 2005). Type I photosystems use iron-sulfur (Fe-S) and quinone reaction centers (RCI) to shuttle the electrons in the photosynthesis electron transport chain, while type II photosystems use pheophytin quinone reaction centers (RCII) to shuttle electrons in photosynthesis (Fig. 3). Also, it is photosystem II that became oxygenic in Cyanobacteria approximately 2.7 to 3.0 billion years ago, by gaining a manganese-assisted protein moiety, which transfers electrons from water into the electron transport chain, and in the process yields oxygen and protons. All other photosystems are anoxygenic, including those in some members of Cyanobacteria. Although there are similar proteins in parts of both PSI and PSII (Fig. 3), there also are significant differences in the orthologs and paralogs, and there are many additional proteins, cofactors, cytochromes, and lipids that are unique to each photosystem. Because each photosystem requires the coordinated expression of a large number of genes, it is unlikely that the duplication of genes from a single photosystem in Cyanobacteria resulted in its complex photosystem. Additionally, gene clusters of the two photosystems are separated within each genome (Beanland 1990; Blankenship 1992; Cardona 2018; Mulkidjanian et al. 2005). Furthermore, RCI and RCII have more similarities to their analogs in other Bacteria than to each other within Cyanobacteria (Cardona 2015, 2016a, b), indicating that the two photosystems diverged in separated taxa prior to being joined in the initial cyanobacterial ancestor.

### Endosymbiosis

Endosymbiotic events resulting from the mixing of hundreds to thousands of genes and gene products that interact successfully are well documented among eukaryotic taxa. They are responsible for the establishment of mitochondrial eukaryotes through one or more endosymbiotic events between members of Archaea and Bacteria, as well as the origin of plastids in plants. They also are responsible for many complex organelles in protists (Archibald and Keeling 2002; Bowler et al. 2009; Ku et al. 2015; Jain et al. 2002; Keeling 2010; Keeling and Palmer 2008; Lake and Rivera 1994; Lang and Gray 1999; Martin and Muller 1998; Poole et al. 1999; Syvanen and Kado 1998). Endosymbiotic events also are consistent with the inheritance of multigene traits, and address the polyphyletic phylogenetic results for membrane systems, photosystems, and other collections of genes in bacteria.

The structure of bacterial cells requires between approximately 100 and 700 genes for their construction and maintenance. For example, in *E. coli* (gamma-Proteobacteria- diderm), more than 650 genes (approximately 16% of the protein-encoding genes) are involved in the construction and maintenance of cell structure (two cell membranes, proteins, and cell walls) (Blattner et al. 1997). *Bacillus subtilis* (Firmicutes - monoderm) has over 100 genes (approximately 2–3% of its protein-encoding genes) dedicated to its cell structure (Kobayashi et al. 2003). Many of these genes are arranged in clusters on the chromosomes. Similarly, photosynthesis in bacteria requires the coordinated expression of more than 100 genes (Beanland 1990; Blankenship 1992; Cardona 2015, 2016a, b, 2018; Mulkidjanian and Junge 1997; Mulkidjanian et al. 2005). While these genes are present in thousands of photosynthetic species, many still exist on contiguous stretches of the genome, suggesting one or a few large HGTs of photosynthetic gene loci have occurred. Endosymbiotic events between a few monoderm taxa are consistent with the mixing of these genomes and present a more parsimonious solution than most other models of bacterial evolution that exclude endosymbiotic events (Gupta 2011, Sousa et al. 2012). When photosystem type and number of membranes are constrained to the fewest number of major events, a minimum of two endosymbiotic events are needed in order to produce a phylogenetic tree in which the photosystems, cell structure, and a number of conserved mutations in a variety of genes are each monophyletic (Figs. 2 and 3; Table 2). The first endosymbiotic event leads to some of the earliest diderms, including the DTF group (*Deinococcus*, *Thermus*, Fusobacteria, and related taxa), as well as the Negativicutes and Halanaerobiales, none of which is capable of photosynthesis. This group of taxa share a mutation that is present in most other phyla of bacteria (Fig. 2; Table 2; Gupta 2003, 2010). One taxon that is often placed close to this group is Thermotogae, which shares the same mutation, but has two additional mutations (a 3–4 aa deletion in the SecF protein, and a 15–17 aa deletion in chorismate synthase). This indicates a divergence after that of Actinobacteria and the DTF group, Negativicutes, and Halanaerobiales. Although Thermotogae often is closest to monoderm taxa, it has sometimes been classified as an atypical diderm. Its outer membrane is unique among bacteria, consisting of mainly protein, thus further separating it from other diderm taxa. The more typical diderms may have been the product of endosymbotic events that occurred long after they had diverged from the line that led to Thermotogae.

### Summary

The parsimonious series of evolutionary events presented here may be part of the reason that a search for a universal tree of life, and identifying the root for this tree, have been difficult. HGT’s have been recognized as a major confounding factor in these studies, and an additional confounder might be the difficulty in accounting for large HGT’s caused by endosymbiotic events. Diderms are polyphyletic and encompass a number of complex characters, including posessing two distinct menbranes and diverse metabolic processes. These factors, plus their antiquity of many evolutionary pathways and branches has led to the myriad contemporary taxa. The composite phylogram (Fig. 2), and pathway (Fig. 3) presented here describe endosymbiotic processes that explain many of the ambiguities in comtemporary morphological and molecular characters present in the bacterial taxa. It also includes the placement of unique taxa that have been alternatively classified as monoderms and diderms (e.g., Thermotogae, Negativicutes, and Halanaerobiales). Consideration of endosymbiotic events may lead to a better understanding of early evolution among bacteria.

## Data Availability

The location of all of the data in this publication are referenced in the text.

## References

[CR1] Antunes LCS, Poppleton D, Klingl A, Criscuolo A, Dupuy B, Brochier-Armanet C, Beloin C, Gribaldo S (2016). Phylogenetic analysis supports the ancestral presence of LPS-outer membranes in the Firmicutes. eLife.

[CR2] Archibald JM, Keeling PJ (2002). Recycled plastids: a ’green movement’ in eukaryotic evolution. Trends Genet.

[CR3] Beanland T (1990). Evolutionary relationships between “Q-type” photosynthetic reaction centres: hypothesis-testing using parsimony. J Theor Biol.

[CR4] Blankenship RE (1992). Origin and early evolution of photosynthesis. Photosynth Res.

[CR5] Blattner FR, Plunkett G, Bloch CA (1997). The complete genome sequence of *Escherichia coli* K-12. Science.

[CR6] Bowler C, Karl DM, Colwell RR (2009). Microbial oceanography in a sea opportunity. Nature.

[CR7] Cardona T (2015). A fresh look at the evolution and diversification of photochemical reaction centers. Photosynth Res.

[CR8] Cardona T (2016). Origin of bacteriochlorophyll *a* and the early diversification of photosynthesis. PLoS ONE.

[CR9] Cardona T (2016). Reconstructing the origin of oxygenic photosynthesis: do assembly and photoactivation recapitulate evolution?. Front Plant Sci.

[CR10] Cardona T (2018). Early Archaean origin of heterodimeric photosystem I. Heliyon.

[CR11] Ciccarelli D, Doerks T, von Mering C, Creevey CJ, Snel B, Bork P (2006). Toward automatic reconstruction of a highly resolved tree of life. Science.

[CR12] Dagan T, Artzy-Randrup Y, Martin W (2008). Modular networks and cumulative impact of lateral transfer in prokaryote genome evolution. Proc Natl Acad Sci USA.

[CR13] Eme L, Spang A, Lombard J, Stairs CW, Ettema TJG (2017). Archaea and the origin of eukaryotes. Nat Rev.

[CR14] Forterre P (2015). The universal tree of life: an update. Front Microbiol.

[CR15] Gao B, Gupta RS (2012). Microbial systematics in the post-genomics era. Antonie Van Leeuwenhoek.

[CR16] Goldfarb KC, Karaoz U, Hanson CA, Santee CA, Brandford MA, Treseder KK, Wallenstein MD, Brodie EL (2011). Differential growth responses of soil bacterial taxa to carbon substrates of varying chemical recalcitrance. Front Microbiol.

[CR17] Gupta RS (1998). Protein phylogenies and signature sequences: a reappraisal of evolutionary relationships among Archaebacteria, Eubacteria, and Eukaryotes. Micobiol Mol Biol Rev.

[CR18] Gupta RS (2003). Evolutionary relationships among photosynthetic bacteria. Photosynth Res.

[CR19] Gupta RS (2010). Molecular signatures for the main phyla of photosynthetic bacteria and their subgroups. Photosynth Res.

[CR20] Gupta RS (2011). Origin of diderm (Gram-negative) bacteria: antibiotic selection pressure rather than endosymbiosis likely led to the evolution of bacterial cells with two membranes. Antonie Van Leeuwenhoek.

[CR21] Gupta RS, Khadka B (2016). Evidence for the presence of key chlorophyll-biosynthesis-related proteins in the genus *Rubrobacter* (Phylum Actinobacteria) and its implications for the evolution and origin of photosynthesis. Photosynth Res.

[CR22] Hug LA, Baker BJ, Anantharaman K, Brown CT, Probst AJ, Castelle CJ, Butterfield CN, Hernsdorf AW, Amano Y, Ise K, Suzuki Y, Dudek N, Relman DA, Finstad KM, Amundson R, Thomas BC, Jillian F (2016). A new view of the tree of life. Nat Microbiol.

[CR23] Imaeda T, Kanetsuna F, Galindo B (1968). Ultrastructure of cell walls of genus *Mycobacterium*. J Ultrastructure Res.

[CR24] Jain R, Rivera MC, Lake JA (1999). Horizontal gene transfer among genomes: the complexity hypothesis. Proc Natl Acad Sci USA.

[CR25] Jain R, Rivera MC, Moore JE, Lake JA (2002). Horizontal gene transfer in microbial genome evolution. Theor Popul Biol.

[CR26] Keeling PJ (2010). The endosymbiotic origin, diversification and fate of plastids. Philos Trans R Soc Lond B.

[CR27] Keeling PJ, Palmer JD (2008). Horizontal gene transfer in eukaryotic evolution. Nat Rev Genet.

[CR28] Kobayashi K, Ehrlich SD, Albertini A (2003). Essential *Bacillus subtilis* genes. Proc Natl Acad Sci USA.

[CR29] Ku C, Nelson-Sathi S, Roettger M, Sousa FL, Lockhart PJ, Bryant D, Hazkani-Covo E, McInerney JO, Landan G, Martin WF (2015). Endosymbiotic origin and differential loss of eukaryotic genes. Nature.

[CR30] Lake JA (2009). Evidence for an early prokaryotic endosymbiosis. Nature.

[CR31] Lake JA, Rivera MC (1994). Was the nucleus the first endosymbiont?. Proc Natl Acad Sci USA.

[CR32] Lang AS, Zhaxybayeva O, Beatty JT (2012). Gene transfer agents: phage-like elements of genetic exchange. Nat Rev Microbiol.

[CR33] Lang BF, Gray MW (1999). Mitochondrial genome evolution and the origin of eukaryotes. Annu Rev Genet.

[CR34] Lebedinsky AV, Chernyh NA, Bonch-Osmolovskaya E (2007). Phylogenetic systematics of microorganisms inhabiting thermal environments. Biochem (Moskow).

[CR35] Macalady JL, Hamilton TL, Grettenberger CL, Jones DS, Tsao LE, Burgos WD (2013). Energy, ecology and the distribution of microbial life. Philos Trans R Soc Lond B Biol Sci.

[CR36] Marin J, Battistuzzi FU, Brown AC, Hedges SB (2016). The timetree of prokaryotes: new insights into their evolution and speciation. Mol Bio Evol.

[CR37] Martin W, Muller M (1998). The hydrogen hypothesis for the first eukaryote. Nature.

[CR38] Mulkidjanian AY, Junge W (1997). On the origin of photosynthesis as inferred from sequence analysis. Photosynth Res.

[CR39] Mulkidjanian AY, Koonin EV, Makarova KS, Kekhedov SL, Sorokin A, Wolf YI, Dufresne A, Partensky F, Burd H, Kaznadzey D, Haselkorn R, Galperin MY (2005). The cyanobacterial genome core and the origin of photosynthesis. Proc Natl Acad Sci USA.

[CR40] Overmann J, Schubert K (2002). Phototropic consortia: model systems for symbiotic interrelations between prokaryotes. Arc Microbiol.

[CR41] Pace N, Sapp J, Goldenfeld N (2012). Phylogeny and beyond: scientific, historical, and conceptual significance of the first tree of life. Proc Natl Acad Sci USA.

[CR42] Poole A, Jeffares D, Penny D (1999). Early evolution: prokaryotes, the new kids on the block. Bioessays.

[CR43] Raymond J, Zhaxybayeva O, Gogarten JP, Gerdes SY, Blankenship RE (2002). Whole-genome analysis of photosynthetic prokaryotes. Science.

[CR44] Sagan L (1967). On the origin of mitosing cells. J Theor Biol.

[CR45] Satoh S, Mimoru M, Tanaka A (2013). Construction of a phylogenetic tree of photosynthetic prokaryotes based on average similarities of whole genome sequences. PLoS ONE.

[CR46] Sousa FL, Shavit-Grievink L, Allen JF, Martin WF (2012). Chlorophyll biogenesis gene evolution indicates photosystem gene duplication, not photosystem merger, at the origin of oxygenic photosynthesis. Genome Biol Evol.

[CR47] Sousy SM, Huang J, Gogarten JP (2015). Horizontal gene transfer: building the web of life. Nat Rev Genet.

[CR48] Syvanen M, Kado CI (1998). Horizontal gene transfer.

[CR49] Tebo BM, Davis RE, Anitori RP, Connell LB, Schiffman P, Staudigel H (2015). Microbial communities in dark oligotrophic volcanic ice cave ecosystems of Mt. Erebus. Antarctica. Front Biol.

[CR50] Wang L, Zhang L, Liu Z, Zhao D, Liu X (2013). A minimal nitrogen fixation gene cluster from *Paenibacillus *sp. WLY78 enables expression of active nitrogenase in *Escherichia coli*. PLoS Genet.

[CR51] Woese CR (1987). Bacterial evolution. Microbiol Rev.

[CR52] Yan Y, Yang J, Dou Y, Chen M (2008). Nitrogen fixation island and rhizosphere competence traits in the genome of root-associated *Pseudomonas stutzeri*. A1501. Proc Natl cad Sci USA.

[CR53] Zeng Y, Feng F, Medova H, Dean J, Koblizek M (2014). Functional type 2 photosynthetic reaction centers found in the rare phylum Gemmatimonadetes. Proc Natl Acad Sci USA.

